# Relationships between vertebral fractures, sex hormones and vitamin D in Moroccan postmenopausal women: a cross sectional study

**DOI:** 10.1186/s12905-015-0199-9

**Published:** 2015-05-13

**Authors:** Aissam El maataoui, Abdellah El Maghraoui, Asmae Biaz, Samira Idrissi Elmachtani, Abdellah Dami, Sanae Bouhsain, Aziza Mounach, Layachi Chabraoui, Zohra Ouzzif

**Affiliations:** University Mohamed V Souissi, Faculty of Medicine and Pharmacy, Av. Mohamed Belarbi El Alaoui Rabat Institut, University Mohamed V Souissi, BP 6203 Rabat, Morocco; Biochemsitry Department, Military Hospital, Rabat, Morocco; Rheumatology Department, Military Hospital, Rabat, Morocco; Biochemistry Department, Ibn Sina Hospital, Rabat, Morocco

**Keywords:** Vertebral fractures, Sex hormones, Vitamin D, VFA, DXA, Bone remodeling markers

## Abstract

**Background:**

Vertebral Fractures (VFs) are associated with bone loss that occurs before menopause but is accelerated at menopause as a result of sex hormone deficiency.

To determine the association of sex hormones, bone remodeling markers and vitamin D levels with bone mineral density (BMD) and asymptomatic VFs prevalence using vertebral fracture assessment (VFA) in a cohort of Moroccan menopausal women.

**Methods:**

This was a cross-sectional study conducted from October 2012 to April 2013 with menopausal women aged 50 years old and over. A total of 207 women who had no previous diagnosis of osteoporosis were enrolled in this cross-sectional study. Women were recruited prospectively from our laboratory department. VFA images and scans of the lumbar spine and proximal femur were obtained using a GE Healthcare Lunar Prodigy densitometer. VFs were defined using a combination of Genant semiquantitative approach and morphometry. Serum levels of estradiol, dehydroepiandrosterone sulfate, Sex hormone binding globulin, vitamin D, Osteocalcin, Crosslaps, intact parathormone were measured by Electrochemiluminescent immunoassay technique.

**Results:**

Among the 207 women, 18.3 % (*n* = 38) had densitometric osteoporosis. On VFA, VFs were detected in 134 (62.3 %), including 96 (44.6 %) grade 1 and 38 (17.6 %) grade 2/3. There was no difference in the plasma levels of sex steroids, bone remodeling markers and vitamin D in the group of women with VFs (grade 1 and grade 2/3) and without VFs. The combination of variables that best predicted grade 2/3 VFs included the number of years since menopause and the lumbar spine T-score.

**Conclusion:**

These data confirm the importance of postmenopausal estrogen and SHBG concentrations in the bone loss and the pathogenesis of osteoporosis in elderly women, but not in the occurrence of the VFs.

## Background

Vertebral fracture (VF) is a serious consequence of osteoporosis that is often under-diagnosed due to the variable clinical presentation and the lack of a gold standard for its definition [1, 2]. Thus, 75 % of VFs are not diagnosed clinically due to the absence of specific symptoms in some cases and the difficulty in determining the cause of these physical symptoms [3]. It has been shown that both symptomatic and asymptomatic VFs are predictors of future osteoporotic fractures [4], and are associated with physical deformity, as well as reduced mobility, quality of life [5, 6], and increased mortality [7, 8]. Also, VFs are associated with bone loss that occurs before menopause but is accelerated at menopause and is the result of sex hormone deficiency [9–11]. Among postmenopausal and elderly women, low estradiol (E2), low testosterone (T), and high sex hormone binding globulin (SHBG) concentrations are associated with a higher risk of hip and non VFs [12, 13]. Dehydroepiandrosterone (DHEA) and its sulfate (DHEAS) are the most abundant circulating steroids. In postmenopausal women during a 15-year follow-up, data suggest that high serum DHEAS at baseline is associated with less bone loss at both femoral neck and lumbar spine and this association diminishes over time [14].

Several studies have shown that sex steroid deficiency is associated with bone loss (REF). However, there is limited data assessing the association between sex hormones and VFs. Thus, the objective of this study was to determine the association of sex hormones, bone remodeling markers and vitamin D levels with bone mineral density (BMD) and prevalence of asymptomatic VFs using vertebral fracture assessment (VFA) in a cohort of Moroccan menopausal women.

## Methods

### Subjects

This was a cross-sectional study conducted from October 2012 to April 2013 with menopausal women 50 years old and over. A total of 207 women aged 50 years and over, who had no previous diagnosis of osteoporosis were recruited prospectively from our laboratory department. General exclusion criteria were non-caucasian origin and diseases, drugs, and other major determinants known to affect bone metabolism. Thus, we excluded subjects with gastrectomy, intestinal resection, recent hyperthyroidism or hyperparathyroidism, recent severe immobilization or treatment with corticosteroids (more than 3 months). Our institutional review board approved this study. The procedures of the study were in accordance with the Declaration of Helsinki, and formal ethics committee approval was obtained for the study (Military Hospital Ethics Committee). All the participants gave an informed and written consent. Each subject completed a standardized questionnaire designed to document putative risk factors of osteoporosis. History of fractures, lifestyle (alcohol consumption, gymnastics or jogging/walking, smoking) and diet (milk, yogurt, cheese) habits were also recorded. The women were asked whether they usually drank milk, coffee, or alcohol, if they ate cheese or yogurt, if they did gymnastics or jogging/walking, and if they smoked tobacco. Menstrual and reproductive histories were assessed: all patients were menopausal since at least 1 year. Height and weight were measured in the rheumatology department before DXA measurement, in light indoor clothes without shoes. Body mass index (BMI) was calculated by dividing weight in kilograms by height in meters squared.

### BMD measurement

Bone mineral density was determined by a Lunar Prodigy Vision DXA system (Lunar Corp., Madison, WI). The DXA scans were obtained by standard procedures supplied by the manufacturer for scanning and analysis. All BMD measurements were carried out by two experienced technicians. Daily quality control was carried out by measurement of a Lunar phantom. At the time of the study, phantom measurements showed stable results. The phantom precision expressed as the coefficient of variation percentage was 0.08. Patient BMD was measured at the lumbar spine (anteroposterior projection at L1–L4) and at the femoral neck. The World Health Organization (WHO) classification system was applied, defining osteoporosis as T-score ≤ −2.5, osteopenia as −2.5 < T-score < −1. Study participants were categorized by the lowest T-score of the L1-4 lumbar spine or femoral neck using our reference values [19]. VFA was classified using the Genant semi quantitative (SQ) approach in the following manner: each VFA image was inspected visually by one expert clinician (AM) to decide whether it contained a fracture in any of the visualized vertebrae and assigned a grade based on the Genant SQ scale [5], where grade 1 (mild) fracture is a reduction in vertebral height of 20–25 %, grade 2 (moderate) a reduction of 26–40 %, and grade 3 (severe) a reduction of over 40 %.

### Biological measurements

All subjects had fasting blood taken in the morning. Samples were left to clot at room temperature for 30 min and then centrifuged. Aliquots of the serum supernatant were frozen and stored at–80 °C and subsequently thawed and analyzed in one batch. Serum estradiol (E2) (reference range, 18.4–201 pmol/l), dehydroepiandrosterone sulfate (S-DHEA) (reference range, 0,33–4,18 μmol/l), Sex hormone binding globulin (SHBG) (reference range, 15–70 ng/ml), vitamin D (25(OH)D3) (reference range, 30–80 ng/ml), Osteocalcin (OC) (reference range, 13–48 μg/l), Crosslaps (β-CTX) (reference range, 0.010–0.854 ng/ml), intact parathormone (PTHi) (reference range, 15–65 pg/ml) were measured by electrochemiluminescent immunoassay (ECLIA) technique (Cobas e601, Roche Diagnostics GmBH, Mannheim, Germany). All the laboratory tests were subject to validation using National External Quality Assurance Schemes. The free estradiol index (FEI) was calculated from the ratio of serum E2 to SHBG.

### Statistical analysis

Results are presented as means (SD) and categorical variables are expressed as frequencies. To compare patients with and without VFs analysis of variance ANOVA was used. Correlations between continuous variables were calculated using Pearson correlation coefficients. Potential risk factors were entered to a stepwise conditional binary regression analysis and the resulted odds ratios with 95 % confidence intervals were reported. The level for significance was taken as *P* ≤ 0.05. Excel 2007 and SPSS 15.0 were used for statistical analysis.

## Results

In this cohort of 207 Caucasian women, the mean (SD) [Range] age was 59.8 (7.6) [50.0–83.0]. Thirty five percent of the patients (*n* = 73) had normal BMD while 46.3 % (*n* = 96) were osteopenic and 18.3 % (*n* = 38) osteoporotic. On vitamin D status, the mean (SD) [range] serum vitamin D concentration for all 207 patients was 13.2 (12.9) ng/mL [3.0–116.5].

**Fig. 1:**
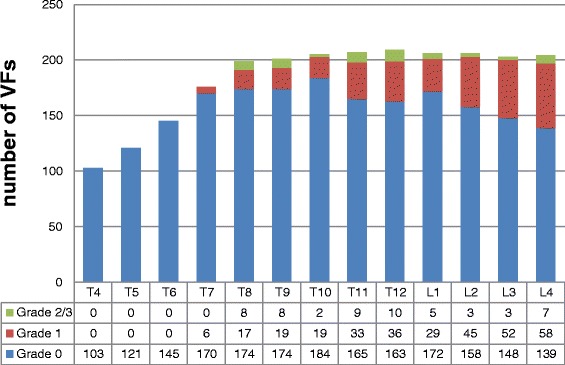
VFA-identified fracture distribution (*n* = 207)

In these 207 women, 88.4 % of vertebrae from T4–L4 and 99.0 % from T8-L4 were adequately visualized on VFA. The percentage of vertebrae not visualized at T4, T5, T6 levels was 42.1 %, 26.9 %, 13.0 % respectively. VFs grades 2/3 were detected in 38 (17.6 %) of these women, while grade 1 were identified in 96 (44.6 %) (Fig. 1).

Comparison between patients without VFs and with grade 1 and grades 2/3 VFs are presented in table 1. Women with grade 2/3 VFs were older and had longer menopause duration. There was no difference in the plasma levels of sex steroids, bone remodeling markers and vitamin D between the three groups.

**Table 1:** Comparison between patients with and without vertebral fractures (*n* = 207)

	Group 0: Women without vertebral fractures (*n* = 73) mean (SD)	Group 1: Women with grade 1 vertebral fractures (*n* = 96) mean (SD)	Group 2: Women with grade 2 or 3 vertebral fractures (*n* = 38) mean (SD)	*P* value	*P* value 0 vs. 2/3	*P* value 0 vs. 1	*P* value 1 vs. 2/3
Age (years)	57,57 (5,92)	60,01 (8,12)	63,64 (8,59)	<0.001	<0.001	0,11	0,04
Body mass index (kg/m^2^)	31,15 (15,54)	31,38 (12,85)	30,25 (5,43)	0,8996	1,00	1,00	1,00
Number of parity	4,78 (1,8)	4,71 (2,5)	5,24 (2,6)	0,49	0,99	1,00	0,72
Years since menopause	7,60 (6,93)	9,40 (8,64)	14,86 (10,83)	<0.001	<0.001	0,55	0,004
SHBG (ng/ml)	52,46 (27,11)	57,87 (28,33)	58,26 (29,75)	0,4044	0,91	0,65	1,00
β-CTX (ng/ml)	0,43 (0,24)	0,43 (0,24)	0,41 (0,24)	0,9159	1,00	1,00	1,00
PTHi (pg/ml)	41,14 (36,83)	39,21 (31,46)	35,89 (5,82)	0,7166	1,00	1,00	1,00
E2 (pmol/l)	47,49 (43,83)	43,42 (51,07)	41,80 (21,70)	0,7689	1,00	1,00	1,00
FEI (pmol/nmol)	12,11 (11,50)	10,64 (18,29)	9,28 (5,94)	0,6026	0,99	1,00	1,00
S-DHEA (μmol/l)	2,34 (1,24)	1,97 (0,98)	2,08 (1,35)	0,1138	0,78	0,12	1,00
25 (OH)D3 (ng/ml)	13,20 (9,54)	12,21 (8,07)	24,42 (3,96)	0,1495	0,41	1,00	0,16
Osteocalcin (μg/l)	24,21 (13,95)	23,83 (12,83)	23,61 (14,33)	0,9722	1,00	1,00	1,00
Lumbar spine BMD (g/cm^2^)	0,97 (0,26)	0,98 (0,18)	1,02 (0,15)	0,4367	0,62	1,00	0,91
Lumbar spine T-score	-1,29 (1,20)	-1,40 (1,55)	-1,09 (1,25)	0,5088	1,00	1,00	0,74
Total hip BMD (g/cm2)	0,93 (0,13)	0,93 (0,14)	0,86 (0,42)	0,2162	0,31	1,00	0,34
Total hip BMD T-score	-0,79 (1,11)	-0,78 (1,20)	-0,71 (1,01)	0,9411	1,00	1,00	1,00

Mean ± SD : m (SD) *: <0.05 **: < 0.001. One way analysis of variance+Post hoc test (Bonferroni) *BMD* bone mineral density, *BMI* body mass index, *E* estradiol, *FEI* free estrogen index, *PTH* parathormone (PTH) intact; *S-DHEA* dehydroepiandrosterone sulphate, *SHBG* sex hormones binding globulin, *T* testosterone

### Correlations of BMD with the clinical and biological parameters

Pearson correlation analysis showed significant negative correlations between the lumbar spine BMD and the following variables: age, number of parity, years since menopause, OC, β-CTX, SHBG and FEI. There was positive correlation between lumbar spine BMD and BMI.

The total hip BMD correlated negatively with the following variables: age, number of parity, years since menopause, OC, β-CTX, SHBG, E2, FEI and S-DHEA. The total hip BMD correlated positively with BMI and S-DHEA (Table 2).

**Table 2:** Correlation between biochemical values and age, BMI and BMD (*n* = 207)

	AGE	BMI	Number of parity	YSM	SHBG (ng/ml)	β-CTX (ng/ml)	PTHi (pg/ml)	E2 (pmol/l)	FEI (pmol/nmol)	S-DHEA (μmol/l)	25 (OH)D3 (ng/ml)	OC (μg/l)
BMI (kg/m2)	-0,117											
Number of parity	0,450^**^	-0,029										
YSM	0,736^**^	-0,079	0,338^**^									
SHBG (ng/ml)	0,183^**^	-0,248^**^	0,024	0,148^*^								
β-CTX (ng/ml)	0,021	-0,127	0,076	-0,039	0,252^**^							
PTHi (pg/ml)	0,024	0,022	0,077	0,026	0,156^*^	0,302^**^						
E2 (pmol/l)	0,023	0,307^**^	0,076	-0,001	-0,016	-0,104	0,125					
FEI (pmol/nmol)	-0,109	0,391^**^	0,039	-0,104	-0,614^**^	-0,252^**^	0,009	0,757^**^				
S-DHEA (μmol/l)	-0,234^**^	0,083	-0,077	-0,147^*^	-0,195^**^	-0,131	-0,133	0,245^**^	0,298^**^			
25(OH)D3 (ng/ml)	-0,043	-0,052	-0,069	0,030	0,155^*^	-0,098	-0,112	0,098	-0,011	0,060		
OC (μg/l)	-0,007	-0,099	-0,041	-0,045	0,277^**^	0,746^**^	0,314**	-0,153^*^	-0,305^**^	-0,203^**^	-0,123	
LS BMD (g/cm^2^)	-0,242^**^	0,293^**^	-0,228^**^	-0,303^**^	-0,292^**^	-0,182^**^	-0,021	0,138^*^	0,320^**^	0,027	0,007	-0,153^*^
LS T-score	-0,244^**^	0,287^**^	-0,233^**^	-0,299^**^	-0,274^**^	-0,186^**^	-0,012	0,133	0,304^**^	0,009	0,008	-0,159^*^
TH BMD (g/cm^2^)	-0,362^**^	0,420^**^	-0,216^**^	-0,389^**^	-0,385^**^	-0,241^**^	-0,083	0,136^*^	0,373^**^	0,160^*^	0,031	-0,277^**^
TH T-score	-0,320^**^	0,410^**^	-0,206^**^	-0,340^**^	-0,362^**^	-0,204^**^	-0,036	0,119	0,337^**^	0,129	0,011	-0,246^**^

*BMD* bone mineral density, *BMI* body mass index, *E* estradiol, *FEI* free estrogen index, *PTH* parathormon, *S-DHEA* dehydroepiandrosterone sulphate, *SHBG* sex hormones binding globulin, *T* testosterone, *YSM* years since menopause

### Correlations of the sex hormones and the SHBG with the clinical parameters

There were positive correlations between the E2 levels and BMI and S-DHEA. There was a negative correlation between E2 and OC. The FEI correlated positively with BMI and S-DHEA, and negatively with β-CTX and OC (Table 2).

There were significant negative correlations between S-DHEA and the following parameters: age, years since menopause, SHBG, and OC. There were significant positive correlations between S-DHEA and E2 and FEI.

There were significant negative correlations between SHBG and BMI, and S-DHEA.

There were significant positive correlations between SHBG and the following parameters: age, β-CTX, 25(OH)D3, years since menopause, and PTHi (Table 2).

### Comparison between patients according to quartiles of E2, FEI and SHBG

Comparison of patients according to quartiles of E2 levels showed that women in the highest quartile had a higher BMI and a higher level of S-DHEA.

Comparison of patients according to quartiles of FEI levels showed that women in the highest quartile had a higher BMI and BMD at the lumbar spine and total hip. They had also the lower prevalence of osteoporosis and β-CTX and OC levels.

Comparison of patients according to quartiles of SHBG levels showed that women in the highest quartiles were older with high prevalence of osteoporosis, and high PTHi, β-CTX levels. They also had a lower lumbar and total hip BMD (Table 3).

**Table 3:** Comparison between patients according to quartiles of E2, FEI and SHBG

E2	Quartile 1	Quartile 2	Quartile 3	Quartile 4	*p*
S-DHEA (μmol/l)	4.699	5.8	10.246	22.92	0.01
BMI (kg/m2)	28,03	29,33	31,465	35,69	0,009
FEI	Quartile 1	Quartile 2	Quartile 3	Quartile 4	*p*
BMI (kg/m2)	28,035	28,135	32,194	36,094	0,002
β-CTX (pg/ml)	0,4785	0,4713	0,3999	0,3537	0,015
OC (μg/l)	27,87	25,35	22,03	20,81	0,024
Lumbar spine BMD (g/cm^2^)	0,9331	0,9288	1,0206	1,0633	0,001
T-score lumbar spine	−1,83	−1,507	−1,122	−0,757	<0,0001
Total hip BMD (g/cm^2^)	0,82031	0,898	0,958	0,9937	<0,0001
T-score total hip	−1,262	−1004	−0,576	−0,251	<0,0001
Prevalence of osteoporosis (%)	54,28	50	17,39	15,21	0,006
SHBG	Quartile 1	Quartile 2	Quartile 3	Quartile 4	*p*
Age (years)	57,27	59,59	61,81	60,81	0,014
β-CTX (pg/ml)	0,3453	0,4271	0,4633	0,4694	0,023
PTHi (pg/ml)	29,83	49,7	43,84	40,11	0,022
Lumbar spine BMD (g/cm^2^)	1,0539	10049	0,9827	0,9014	0,02
T-score lumbar spine	−0,843	−1,204	−1,435	−1,753	0,045
Total hip BMD (g/cm^2^)	1,00609	0,93952	0,83704	0,8866	<0,0001
Total hip BMD T-score	−0,185	−0,689	−1,072	−1,164	<0,0001
Prevalence of osteoporosis (%)	14,89	22,72	45,94	51,42	0,026

### Multiple logistic regression

Significant variables in the univariate analysis were entred in a stepwise logistic regression analysis for the presence of grade 2/3 VFs (grade 1 VFs were excluded from this analysis) to determine the combination of variables that best predicted grade 2/3 vertebral fractures in postmenopausal women over 50 years. The best model included the number of years since menopause and the lumbar spine T-score (Table 4).

**Table 4:** Stepwise logistic regression analysis for the presence of grade 2/3 vertebral fractures

	Exp (B)	95 % Confidence interval		*P*
		Lower	Higher	
Years since menopause: mean (SD)	1,079	1,036	1,123	<0.001
Lumbar spine T-score: mean (SD)	1,558	1,112	2,184	0,01

## Discussion

Our study showed that women with grade 2/3 VFs were older and had higher number of years since menopause. The best model that predicts grade 2/3 VFs comprised the number of years since menopause and the lumbar spine T-score. There was no difference in the plasma levels of sex steroids, bone remodeling markers and vitamin D.

VFs were indentified using VFA in 134 (62.3 %), including 96 (44.6 %) grade 1 and 38 (17.67 %) grade 2/3. This prevalence of VFs in our population (grade 2/3) is similar to figures reported in western Caucasian population [15]. Prevalence rates varied between 19 % and 24 % in elderly women from the Middle East, Europe and North America [16]. In a Moroccan study VFs grade 2/3 was detected in 25.6 % of women (17.6 % of women in this study) [17]. The main difference between these two Moroccan cohorts was the mean age of participants. Indeed, in the cohort of El Maghraoui et al. the patients were older (mean age 65 years) and 43 % of women were osteoporotic [17], whereas in this study, the patients were younger (mean age 59) with a higher BMI and only 18 % were osteoporotic.

Comparison of patients according to quartiles of E2 levels showed that women in the highest quartile had a higher BMI and a higher level of S-DHEA. Pearson correlation analysis showed that there were positive correlations between E2 and BMI and S-DHEA. There was a negative correlation between the E2 and OC. The positive correlation between E2 and BMI is explained by the relationship between estrogen levels and aromatisation of estrogen precursors in fat stores [18]. The positive association between E2 and S-DHEA, may be related to the fact that S-DHEA is an inactive adrenal precursor that is metabolized into active androgens and estrogens in peripheral tissues.

In contrast to the OFELY study, we did not find any association between E2 and VFs. This may be due to the lower mean age of women in our study compared to the OFELY study (67 years in women with fracture and 64 in control) [19]. Indeed, in the OFELY study, women with serum levels of E2 and DHEAS in the lowest quartile had an RR of fracture (20 vertebral and 35 peripheral fractures) of 2.2 (1.2-4.0) and 2.1 (1.2-3.8) respectively [19]. Findings from the Study of Osteopororic Fracture (SOF) suggest that women with estradiol levels > 10 pg/ml averaged only 0.1 % (−0.7 %, 0.5 %) annual hip bone loss while women with levels below 5 pg/ml averaged 0.8 % (0.3, 1.2) hip bone loss per year [20]. In a large prospective cohort of 7598 healthy elderly ambulatory women (EPIDOS study), aged 75 years or more. women having serum E2 in the highest quartile (i.e., >10 pg/ml) were protected, with an HR of 0.66 (0.44–0.98) that did not remain significant after adjustment for weight (HR = 0.71 [0.47–1.06]) [21].

In our study we found a negative correlation between the SHBG and the BMD at the lumbar spine and the total hip. And women in the highest quartiles of SHBG were older with greater prevalence of osteoporosis and high PTH_i_ and β-CTX levels. But we did not find any association between SHBG and the prevalence of VFs. In contrast, several studies have found that high SHBG levels were associated with the subsequent occurrence of VFs, even after adjustment for age and body weight, with odds ratios of about 1.55 [22–24]. Hoppe and al suggest that the SHBG level may be a marker for the severity of osteoporosis, because they found in a study of 184 osteoporotic women having a mean age of 76 years, that plasma SHBG levels were associated with the presence of one or more VFs and with the number of prevalent fractures [25].

In the lowest quartile of the FEI, we have a significant increase in both OC and β-CTX in comparison to the highest quartile of SHBG in which only an increase in OC level was detected. Also, the decrease in BMD at the lumbar spine is more important at the lowest quartile of SHBG compared to the highest quartile of SHBG. These findings support that in addition to its action on bioavailability of sex hormones, there is a new pathway by which sex steroids may act on osteoblasts. The sex steroid/SHBG complex binds to the specific SHBG receptor (SHBG-R), thereby activating the intracellular signal transduction pathways [26].

Devine et al. have proved that incident fracture was predicted by the FEI after adjustment for age, weight, use of topical estrogen, calcium supplementation and prevalent fracture. The risk of VF increased with decreased free estradiol index (HR per SD reduction: 1.63:95 % CI: 0.91–2.92) [27]. However, a later study indicated that the effect of free estradiol on fracture risk in women was attenuated when adjusted for testosterone and SHBG levels and no longer evident when further adjusted for body mass index [12]. Similarly, a study in a French population showed that the association of higher free estradiol with a lower risk of hip fracture was no longer significant after adjustment for weight [21]. In addition to estrogen several other factors have been implicated in bone strength, factors such as progesterone deficiency [28], and polymorphisms of genes of the receptor activator of NFκB ligand signaling system [29]. Also, the ERα is the predominant subtype in postmenopausal bone specimens [30], and there is evidence that mice with normal ERα, but non-functional ERβ, have normal BMD and trabecular structure [31].

DHEA is an inactive adrenal precursor that is metabolized into active androgens and estrogens in peripheral tissues. We found significant negative correlations between S-DHEA and age, years since menopause, SHBG, and OC. Also, we found significant positive correlations between S-DHEA and E2 and FEI. However, we did not find any association between the level of S-DHEA and the prevalence of VFs. The positive association of S-DHEA to estradiol and free estradiol may be related to metabolization of S-DHEA into active androgens and estrogens in peripheral tissues. The negative associations of DHEA-S to estradiol and free estradiol can be explained by increasing age of patients. In fact, increasing age is associated to the decrease in DHEA-S and consequently the decrease in the levels of androgens and estrogens.

On vitamin D status, the mean ± SD [range] serum vitamin D concentration for all 207 patients was 13,23 ± 12,96 ng/mL [3,00 -116,50], taking into account that the study was conducted in winter. Indeed, in a Moroccan study, Allali and al. found that the main determinants of hypovitaminosis D were age > 55 years [OR 2.14 (95 % IC, 1.1-4.1; =0.026)], wearing a veil [OR 2 (95 % IC, 1.1-4; *P* = 0.04)], time spent outdoors less than 30 min/day [OR 2.8 (95 % IC: 1.4-5.7; *P* = 0.003)], and daily calcium intake less than 700 mg [OR 2.39 (95 % IC.1.2-4.7; *P* < 0.01)] [32].

On the association between VFs and vitamin D status the results of previous studies are contradictory. In our study, there was no difference in the plasma level of vitamin D in the group of women with and without VFs (grade 1 and grade 2/3). May be because of the mean age of the participants in these study was low, and for younger patients vitamin D status may play a less important role in fracture risk. Lopes et al. evaluating the incidence of VFs observed similar results [33] while, in contrast to our study, other authors observed significant association with vitamin D insufficiency [34, 35].

Our study has strengths and limitations. All of the DXA and biochemical measurements were conducted with a single bone densitometer and a single biochemistry laboratory, with very careful quality controls in place. All the morphometric assessments were made by an experienced investigator. Before diagnosis of fracture, a non-osteoporotic origin was considered for each deformity. However, even history of trauma was inquired, we cannot exclude that some subjects did not report remote traumas. The main limitations lie in the cross-sectional nature of the study and in the procedures used to select subjects, who were all volunteers and ambulatory.

## Conclusion

In summary, we have shown that the best model that predicts grade 2/3 VFs comprised the number of years since menopause and the BMD of the lumbar spine. VFs are the consequence of the decrease of bone mass and bone strength with age and duration of menopause (decline in the concentration of estrogens), this findings support the view that aging is the pivotal determinant of bone mass and bone strength [36]. We did not find any association between sex hormones and the prevalence of VFs, likely because of the low mean age of the studied women (59.8 years). However, the decreased levels of FEI and the increased levels of SHBG were associated with increased bone turnover markers and low BMD. These data confirm the important role played by postmenopausal estrogen deficiency and SHBG concentrations in the pathogenesis of osteoporosis of elderly women. For VFs, we must take into account other factors involved in the quality of the bone strength.

## Abbreviations

BMD: Bone mineral density
VFs: Vertebral fractures
VFA: Vertebral fracture assessment
DHEA: Dehydroepiandrosterone
DHEAS: Dehydroepiandrosterone sulfate
BMI: Body mass indexE2
E2: Estradiol
SHBG: Sex hormone binding globulin
25(OH)D3: Vitamin D
OC: Osteocalcin
β-CTX: Crosslaps
PTHi: Intact parathormone
FAI: Free androgen index
FEI: Free estradiol index

## References

[1] Cummings SR, Melton LJ (2002). Epidemiology and outcomes of osteoporotic fractures. Lancet.

[2] Delmas PD, van de Langerijt L, Watts NB, Eastell R, Genant H, Grauer A (2005). Underdiagnosis of vertebral fractures is a worldwide problem: the IMPACT study. J Bone Miner Res.

[3] Pasco JA, Henry MJ, Korn S, Nicholson GC, Kotowicz MA (2009). Morphometric vertebral fractures of the lower thoracic and lumbar spine, physical function and quality of life in men. Osteoporos Int.

[4] Delmas PD, Genant HK, Crans GG, Stock JL, Wong M, Siris E (2003). Severity of prevalent vertebral fractures and the risk of subsequent vertebral and nonvertebral fractures: results from the MORE trial. Bone.

[5] Ettinger B, Pressman A, Sklarin P, Bauer DC, Cauley JA, Cummings SR (1998). Associations between low levels of serum estradiol, bone density, and fractures among elderly women: the study of osteoporotic fractures. J Clin Endocrinol Metab.

[6] Nevitt MC, Ettinger B, Black DM, Stone K, Jamal SA, Ensrud K (1998). The association of radiographically detected vertebral fractures with back pain and function: a prospective study. Ann Intern Med.

[7] Ensrud KE, Thompson DE, Cauley JA, Nevitt MC, Kado DM, Hochberg MC (2000). Prevalent vertebral deformities predict mortality and hospitalization in older women with low bone mass. Fracture Intervention Trial Research Group. J Am Geriatr Soc.

[8] Kado DM, Browner WS, Palermo L, Nevitt MC, Genant HK, Cummings SR (1999). Vertebral fractures and mortality in older women: a prospective study. Study of Osteoporotic Fractures Research Group. Arch Intern Med.

[9] Emaus N, Berntsen GK, Joakimsen R, Fonnebo V (2006). Longitudinal changes in forearm bone mineral density in women and men aged 45–84 years: the Tromso Study, a population-based study. Am J Epidemiol.

[10] Riggs BL, Khosla S, Melton LJ (1998). A unitary model for involutional osteoporosis: estrogen deficiency causes both type I and type II osteoporosis in postmenopausal women and contributes to bone loss in aging men. J Bone Miner Res.

[11] Slemenda C, Longcope C, Peacock M, Hui S, Johnston CC (1996). Sex steroids, bone mass, and bone loss. A prospective study of pre-, peri-, and postmenopausal women. J Clin Investig.

[12] Lee JS, LaCroix AZ, Wu L, Cauley JA, Jackson RD, Kooperberg C (2008). Associations of serum sex hormone-binding globulin and sex hormone concentrations with hip fracture risk in postmenopausal women. J Clin Endocrinol Metab.

[13] Bjornerem A, Ahmed LA, Joakimsen RM, Berntsen GK, Fonnebo V, Jorgensen L (2007). A prospective study of sex steroids, sex hormone-binding globulin, and non-vertebral fractures in women and men: the Tromso Study. Eur J Endocrinol.

[14] Ghebre MA, Hart DJ, Hakim AJ, Kato BS, Thompson V, Arden NK (2011). Association between DHEAS and bone loss in postmenopausal women: a 15-year longitudinal population-based study. Calcif Tissue Int.

[15] Johnell O, Kanis J (2005). Epidemiology of osteoporotic fractures. Osteoporos Int.

[16] Ballane G, Cauley JA, Arabi A, El-Hajj Fuleihan G, Cauley RMFWDLA (2013). Chapter 27 - Geographic Variability in Hip and Vertebral Fractures. Osteoporosis.

[17] El Maghraoui A, Morjane F, Nouijai A, Achemlal L, Bezza A, Ghozlani I (2009). Vertebral fracture assessment in Moroccan women: prevalence and risk factors. Maturitas.

[18] Simpson ER, Bulun SE, Nichols JE, Zhao Y (1996). Estrogen biosynthesis in adipose tissue: regulation by paracrine and autocrine mechanisms. J Endocrinol.

[19] Garnero P, Sornay-Rendu E, Claustrat B, Delmas PD (2000). Biochemical markers of bone turnover, endogenous hormones and the risk of fractures in postmenopausal women: the OFELY study. J Bone Miner Res.

[20] Stone K, Bauer DC, Black DM, Sklarin P, Ensrud KE, Cummings SR (1998). Hormonal predictors of bone loss in elderly women: a prospective study. The Study of Osteoporotic Fractures Research Group. J Bone Miner Res.

[21] Chapurlat RD, Garnero P, Breart G, Meunier PJ, Delmas PD (2000). Serum estradiol and sex hormone-binding globulin and the risk of hip fracture in elderly women: the EPIDOS study. J Bone Miner Res.

[22] van Hemert AM, Birkenhager JC, De Jong FH, Vandenbroucke JP, Valkenburg HA (1989). Sex hormone binding globulin in postmenopausal women: a predictor of osteoporosis superior to endogenous oestrogens. Clin Endocrinol (Oxf).

[23] Tromp AM, Ooms ME, Popp-Snijders C, Roos JC, Lips P (2000). Predictors of fractures in elderly women. Osteoporos Int.

[24] Goderie-Plomp HW, van der Klift M, de Ronde W, Hofman A, de Jong FH, Pols HA (2004). Endogenous sex hormones, sex hormone-binding globulin, and the risk of incident vertebral fractures in elderly men and women: the Rotterdam Study. J Clin Endocrinol Metab.

[25] Hoppé E, Bouvard B, Legrand E, Levasseur R, Masson C, Audran M: Serum Sex Hormone-Binding Globulin Level to Predict Osteoporosis Severity. In: Journal of Bone and Mineral Research 2008: Amer Soc Bone & Mineral Res, Washington, DC 20036-3309 USA; 2008: S447-S447.

[26] Hoppe E, Bouvard B, Royer M, Audran M, Legrand E (2010). Sex hormone-binding globulin in osteoporosis. Joint Bone Spine.

[27] Devine A, Dick IM, Dhaliwal SS, Naheed R, Beilby J, Prince RL (2005). Prediction of incident osteoporotic fractures in elderly women using the free estradiol index. Osteoporos Int.

[28] Seifert-Klauss V, Prior JC (2010). Progesterone and bone: actions promoting bone health in women. J Osteoporos.

[29] Dong SS, Liu XG, Chen Y, Guo Y, Wang L, Zhao J (2009). Association analyses of RANKL/RANK/OPG gene polymorphisms with femoral neck compression strength index variation in Caucasians. Calcif Tissue Int.

[30] Batra GS, Hainey L, Freemont AJ, Andrew G, Saunders PT, Hoyland JA (2003). Evidence for cell-specific changes with age in expression of oestrogen receptor (ER) alpha and beta in bone fractures from men and women. J Pathol.

[31] Nilsson S, Gustafsson JA (2011). Estrogen receptors: therapies targeted to receptor subtypes. Clin Pharmacol Ther.

[32] Allali F, El Aichaoui S, Khazani H, Benyahia B, Saoud B, El Kabbaj S (2009). High prevalence of hypovitaminosis D in Morocco: relationship to lifestyle, physical performance, bone markers, and bone mineral density. Semin Arthritis Rheum.

[33] Lopes JB, Danilevicius CF, Takayama L, Caparbo VF, Scazufca M, Bonfá E (2009). Vitamin D insufficiency: a risk factor to vertebral fractures in community-dwelling elderly women. Maturitas.

[34] El Maghraoui A, Ouzzif Z, Mounach A, Rezqi A, Achemlal L, Bezza A (2012). Hypovitaminosis D and prevalent asymptomatic vertebral fractures in Moroccan postmenopausal women. BMC Womens Health.

[35] Cummings SR, Browner WS, Bauer D, Stone K, Ensrud K, Jamal S (1998). Endogenous hormones and the risk of hip and vertebral fractures among older women. N Engl J Med.

[36] Hui SL, Slemenda CW, Johnston CC (1988). Age and bone mass as predictors of fracture in a prospective study. J Clin Invest.

